# The pathophysiology of rapid fluctuations in mental status associated with olanzapine: A case report

**DOI:** 10.3389/fpsyt.2022.1028350

**Published:** 2022-12-01

**Authors:** Tyler J. Torrico

**Affiliations:** ^1^Department of Psychiatry, Kern Medical, Bakersfield, CA, United States; ^2^American Psychiatric Association Substance Abuse and Mental Health Services Administration (SAMHSA) Minority Fellowship, Washington, DC, United States

**Keywords:** CYP: cytochrome P450, (H_1_): histamine H_1_ receptor, (M_1_): muscarinic M_1_ receptor, adverse (side) effects, drug induced abnormalities, delirium, antipsychotic, schizophrenia

## Abstract

**Background:**

Olanzapine toxicity is reported to be a rare but specific phenomenon characterized by rapid fluctuations between somnolence and agitation, which has been referred to as “agitation despite sedation.” A similar phenomenon is observed as an adverse reaction of the long-acting injectable olanzapine formulation, which has been referred to as “delirium/sedation syndrome.”

**Case presentation:**

This case report describes a 48-year-old man diagnosed with schizophrenia who experienced rapid fluctuations between somnolence and agitation during a cross-titration of olanzapine to clozapine. The patient had normal serum levels of both medications and the symptoms resolved with the discontinuation of olanzapine.

**Conclusion:**

Rapid fluctuations in mental status between somnolence and agitation are not clearly described among other antipsychotics, and it is possible that this phenomenon may be specific to olanzapine. The findings of this case report suggested that this phenomenon was likely the result of the oversaturation of (H_1_) and (M_1_) receptors.

## Introduction

“Agitation despite sedation” is a rare complication in the setting of olanzapine toxicity characterized by rapid fluctuations between somnolence and agitation (1). This clinical presentation is similar to “post-injection delirium/sedation syndrome,” a known adverse reaction from the long-acting injectable (LAI) formulation of olanzapine. “Agitation despite sedation” has been described in cases of overdose and “post-injection delirium/sedation syndrome” is hypothesized to occur after there is vessel injury from the injection process, leading to the unintentional intravascular entry of LAI dose (2). Both of these events appear to occur when there is a high concentration of serum olanzapine, leading to the clinically similar but separately named phenomenon.

Clinicians often resort to using two or more antipsychotics in treatment-resistant schizophrenia, although this practice is controversial and complex. The use of two or more antipsychotics is referred to as antipsychotic polypharmacy (APP) and may be an appropriate strategy in some clinical scenarios such as active cross-titration or augmentation with aripiprazole for negative symptom reduction (3). Although APP generally lacks double-blind or high-quality evidence of efficacy, some patients can benefit from APP without further negative consequences (3). However, for some patients, there are still significant concerns for APP practice including increased risk of acute and long-term side effects, toxicity, and potential for drug–drug interactions (4).

## Case presentation

A 48-year-old Caucasian man with a past medical history of chronic active hepatitis C, anisocoria, and long-standing history of treatment-resistant schizophrenia was referred for inpatient psychiatric hospitalization (at a non-smoking facility) after experiencing worsening psychosis in the context of recent methamphetamine use and self-discontinuation of olanzapine. Prior to the admission, the patient was smoking approximately 10 cigarettes daily. Given the prior history of therapeutic response to olanzapine, the patient was started on olanzapine 5 mg/day. Olanzapine was titrated to 40 mg/day on day 6 of hospitalization, which was chosen due to moderate evidence for efficacy at this dose supported by a double-blind, controlled clinical trial in patients with severe/persistent psychotic symptoms (5). On day 12, the patient continued to suffer from auditory hallucinations and disorganized (but not violent) thought, speech, and behavior and was thereafter planned for cross-titration to clozapine due to persistent treatment-resistant psychosis on high-dose olanzapine. The patient received clozapine 12.5 mg with plans to titrate it to a therapeutic dose before tapering olanzapine. The patient received no other psychotropic medications during this hospitalization.

Clozapine continued to be titrated up to 250 mg/day with a reduction of olanzapine dose to 30 mg/day in the hospital on day 22 and with the patient clinically tolerating the psychotropic regimen up to this point. In the early morning of day 23, the patient had rapidly fluctuating mental status between somnolence and agitation. Specifically, the patient had rapid fluctuations between sleep with loud snoring and violent agitation within 20–30 s of intervals. The patient’s presentation was concerning for an episode of psychotic-related agitation, and the patient was administered 50 mg of chlorpromazine one time, with no significant improvement. However, as the night progressed, symptoms resolved, and the patient did not have similar relapsing symptoms until the following night. Overnight in the hospital on day 24, the patient continued to exhibit rapid fluctuations between somnolence and agitation. The severity of agitation required 4-point restraints for safety, and the patient received 25 mg of intramuscular chlorpromazine and later 2 mg of oral lorazepam with no therapeutic response. The patient was transferred to the internal medicine service for the evaluation of encephalopathy. Differential diagnoses at the time included neuroleptic malignant syndrome, catatonia, acute medical illness (i.e., acute hepatitis or SARS-CoV-2 infection), and medication-induced delirium.

During these episodes, the patient’s cognition was tested and noted to have significantly deviated from his baseline. The patient had impaired short-term memory, disorientation, reduced attention, and concentration. Orthostatic vital signs were within normal limits. Physical exam was negative for clonus, hyperreflexia, muscle rigidity, or signs of catatonia. Miosis was not appreciated outside of the patients’ previously documented anisocoria. An extensive medical workup to investigate the causes of altered mental status was completed with unremarkable findings for the following: complete blood count (CBC), complete metabolic count (CMP), ammonia, thyroid studies, creatinine kinase, troponins, urinalysis, urine culture, urine toxicology, SARS-CoV-2 reverse transcription polymerase chain reaction (RT-PCR), human immunodeficiency virus antibody/antigen 4th generation testing, syphilis antibody ELISA testing, electrocardiogram, chest X-ray, and non-contrast head computed tomography (CT). The patient was unable to have a brain magnetic resonance imaging (MRI) completed due to having metal in his body from a prior gunshot wound. Lumbar puncture revealed normal opening pressure, mild lymphocytic pleocytosis (white blood cells 6/μl), normal glucose (48 mg/dl), and elevated protein level (70.8 g/dl). The autoimmune encephalitis evaluation CSF panel (anti-NMDA receptor, anti-LGI1, anti-GAD65, anti-GABA-B, anti-CASPR2, and anti-AMPA-R) of the University of Pennsylvania showed negative results.

For the next 3 days, the patient was continued on the same dose of medications as there was initially low suspicion for medication-induced delirium as the patient had previously clinically tolerated clozapine 250 mg/day with olanzapine 30 mg/day. Furthermore, clozapine serum level was 415 mg/L (reference 350–600 ng/ml), norclozapine level was 116 mg/L, and olanzapine level was 38.6 ng/ml (reference 20–40 ng/ml, toxic > 80 ng/ml) [5,6]. An electroencephalogram (EEG) conducted for 1 h showed a mild decline in diffusion but no seizures or epileptic discharges. The patient developed new mild transaminitis (ALT 114 unit/L and AST 55 unit/L), hepatitis C viral load returned to 2,120,000 RNA copies/mL (consistent with previous levels), and liver ultrasound was unremarkable. Genetic testing for cytochrome P450 (CYP) metabolism indicated the patient had CYP1A2 genotype of *1F/*1F (phenotype for rapid metabolizer). These findings suggested a concern for antipsychotic toxicity secondary to altered medication metabolism, and olanzapine was discontinued.

The day after olanzapine was stopped, the patient had significant clinical improvement including full orientation, improved concentration, and attention, but still with auditory hallucinations. The patient thanked the treatment teams for his return to his baseline state of cognition and reported that he physically felt much better after olanzapine was discontinued. For the schizophrenia of patient, he was able to return to the behavioral health unit, and no adjustments of clozapine were indicated for the remainder of hospitalization. Outpatient evaluation 6 months after hospital discharge, the patient continued to tolerate clozapine 250 mg/day with no adverse reactions.

## Discussion

This case report describes a patient with schizophrenia treated with an APP regimen of olanzapine and clozapine who experienced rapid fluctuations in mental status, which was clinically similar to previous descriptions of “agitation despite sedation” and “post-injection delirium/sedation syndrome” (1, 2). Of interest, both serum olanzapine and clozapine levels were within therapeutic ranges well below the threshold for toxicity (80 ng/ml and 600 mcg/L, respectively) (6–8). Therefore, the symptoms observed in this case are not likely to be explained by olanzapine or clozapine toxicity alone, especially considering the absence of classic clozapine toxicity symptoms such as sialorrhea, ataxia, and convulsions (9). Due to the rare and broad clinical presentation of clozapine-induced myocarditis, cardiac evaluation should be investigated in all patients with an acute adverse decline in physical health (10). Notably, the patient’s symptoms resolved with the discontinuation of olanzapine, while the clozapine dose was able to be maintained throughout hospitalization.

Clozapine and olanzapine have multiple similarities including molecular structure and major metabolism through CYP 1A2. It appears likely that the combined off-target effects for both medications were additive, especially considering that both have similar (but not equal) potency at multiple neuroreceptors (Table 1) (11). Compared to typical antipsychotics, both olanzapine and clozapine share relatively weaker antagonism to dopamine (D_2)_ receptors and stronger antagonism to serotonin (5-HT_2A)_ receptors. Both of these agents, especially olanzapine, have high binding affinities to histamine (H_1_) receptors, which are centrally acting and, in cases of toxicity, can precipitate central nervous system depression and sedation (1, 11). Furthermore, both have potent muscarinic (M_1_) antagonism which is likely primarily responsible for delirium and agitation (1, 11).

**TABLE 1:** Data are represented by the equilibrium constant (Ki).

	H_1_	M_1_
**Off-target antipsychotic receptor binding**		
Olanzapine	0.08	2.5
Clozapine	3.1	1.4
Quetiapine	19	120
Risperidone	5.2	>10,000
Haloperidol	260	>10,000
Adverse reaction of interest	Sedation	Delirium (agitation, memory, and cognitive impairment)

Values represent the nanomolar amount required to block 50% of the specified receptor. Pharmacokinetically, the lower the equilibrium constant (Ki), the stronger a receptor is bound (11).

Patient-specific changes in CYP 1A2 metabolism may contribute to pharmacologic toxicity. In this case, the genetic testing revealed a CYP1A2 genotype of *1F/*1F, which would be expected to increase drug metabolism. However, the clozapine-to-norclozapine ratio obtained was 3.58, while the patient was symptomatic, which is indicative of a significant decrease in drug metabolism (a ratio of > 2 suggests metabolism inhibition or saturation) (12). This suggests a superimposed acquired CYP dysfunction as the patient’s phenotype would be expected to have a much lower clozapine-to-norclozapine ratio. The patient had new mild transaminitis but did not have significant changes in hepatitis C viral load or abnormality in liver ultrasound, suggesting liver injury unrelated to hepatitis C. Of note, increases in serum transaminases are a common adverse reaction of clozapine and may explain the mild transaminitis observed in this patient (13). Cigarette smoke is an inducer of CYP 1A2; there have been several reported cases of severe adverse effects associated with elevated serum levels of olanzapine or clozapine in the setting of smoking discontinuation (14, 15). The patient described in this case report began to experience rapid fluctuations in mental status 3 weeks after medication initiation and smoking discontinuation, which correlates with the estimated CYP 1A2 turnover time of 2 weeks (14).

## Conclusion

Rapid fluctuations in mental status between somnolence and agitation are not clearly described among other antipsychotics, and it is possible that “agitation despite sedation” and “delirium/sedation syndrome” may be specific to olanzapine (1, 2). The findings of this case report suggested that this phenomenon is likely the result of the oversaturation of (H_1_) and (M_1_) receptors. This was evidenced by clozapine and olanzapine having similar neuroreceptor affinity and a lack of toxic serum levels individually. Early recognition of rapid fluctuating mental status between somnolence and agitation should alert clinicians to a possible medication-induced delirium from saturated (H_1_) and (M_1_) antagonism, particularly in patients with risk factors for alterations in CYP metabolism.

## Data availability statement

The original contributions presented in this study are included in the article/supplementary material, further inquiries can be directed to the corresponding author/s.

## Ethics statement

The studies involving human participants were reviewed and approved by the Kern Medical Institutional Review Board. The patients/participants provided their written informed consent to participate in this study.

## Author contributions

The author confirms being the sole contributor of this work and has approved it for publication.

## Abbreviations

APP: antipsychotic polypharmacy
CYP: cytochrome P450
mg: milligrams
CBC: complete blood count
CMP: complete metabolic panel
ECG: electrocardiogram
CT: computed tomography
EEG: electroencephalogram
CSF: cerebrospinal fluid
(H_1_): histamine H1 receptor
(M_1_): muscarinic M1 receptor
RT-PCR: reverse transcription polymerase chain reaction
CT: computed tomography
MRI: magnetic resonance imaging.
